# D-Amphetamine Rapidly Reverses Dexmedetomidine-Induced Unconsciousness in Rats

**DOI:** 10.3389/fphar.2021.668285

**Published:** 2021-05-18

**Authors:** Risako Kato, Edlyn R. Zhang, Olivia G. Mallari, Olivia A. Moody, Kathleen F. Vincent, Eric D. Melonakos, Morgan J. Siegmann, Christa J. Nehs, Timothy T. Houle, Oluwaseun Akeju, Ken Solt

**Affiliations:** ^1^ Department of Anesthesia, Critical Care and Pain Medicine, Massachusetts General Hospital, Boston, MA, United States; ^2^ Department of Anaesthesia, Harvard Medical School, Boston, MA, United States; ^3^ University of Massachusetts Medical School, Worcester, MA, United States; ^4^ Institute of Medical Engineering and Science, Massachusetts Institute of Technology, Cambridge, MA, United States

**Keywords:** dexmedetomidine, ketamine, d-amphetamine, prefrontal cortex, emergence from anesthesia

## Abstract

D-amphetamine induces emergence from sevoflurane and propofol anesthesia in rats. Dexmedetomidine is an α_2_-adrenoreceptor agonist that is commonly used for procedural sedation, whereas ketamine is an anesthetic that acts primarily by inhibiting NMDA-type glutamate receptors. These drugs have different molecular mechanisms of action from propofol and volatile anesthetics that enhance inhibitory neurotransmission mediated by GABA_A_ receptors. In this study, we tested the hypothesis that d-amphetamine accelerates recovery of consciousness after dexmedetomidine and ketamine. Sixteen rats (Eight males, eight females) were used in a randomized, blinded, crossover experimental design and all drugs were administered intravenously. Six additional rats with pre-implanted electrodes in the prefrontal cortex (PFC) were used to analyze changes in neurophysiology. After dexmedetomidine, d-amphetamine dramatically decreased mean time to emergence compared to saline (saline:112.8 ± 37.2 min; d-amphetamine:1.8 ± 0.6 min, *p* < 0.0001). This arousal effect was abolished by pre-administration of the D_1_/D_5_ dopamine receptor antagonist, SCH-23390. After ketamine, d-amphetamine did not significantly accelerate time to emergence compared to saline (saline:19.7 ± 18.0 min; d-amphetamine:20.3 ± 16.5 min, *p* = 1.00). Prefrontal cortex local field potential recordings revealed that d-amphetamine broadly decreased spectral power at frequencies <25 Hz and restored an awake-like pattern after dexmedetomidine. However, d-amphetamine did not produce significant spectral changes after ketamine. The duration of unconsciousness was significantly longer in females for both dexmedetomidine and ketamine. In conclusion, d-amphetamine rapidly restores consciousness following dexmedetomidine, but not ketamine. Dexmedetomidine reversal by d-amphetamine is inhibited by SCH-23390, suggesting that the arousal effect is mediated by D_1_ and/or D_5_ receptors. These findings suggest that d-amphetamine may be clinically useful as a reversal agent for dexmedetomidine.

## Introduction

Patients emerging from general anesthesia can present numerous challenges including airway and oxygenation problems (Fasting and Gisvold, 2002), hemodynamic instability (Miyazaki et al., 2009), and delirium (Mason, 2017). In addition, delayed emergence often has a negative impact on operating room efficiency and sometimes presents a diagnostic quandary (Tzabazis et al., 2015). It has also been reported that delayed emergence increases the risk for postoperative delirium and cognitive dysfunction in elderly patients (Robinson et al., 2009; Brown and Deiner, 2016). In current practice, emergence from general anesthesia is dictated by the pharmacokinetics of drug clearance, leaving anesthesia providers with limited control over the arousal states of patients. Thus, novel strategies to safely expedite the recovery of consciousness in anesthetized patients may help to overcome problems associated with anesthetic emergence.

D-amphetamine is commonly prescribed in oral form for the treatment of Attention Deficit Hyperactivity Disorder, and acts by directly releasing norepinephrine and dopamine from presynaptic terminals (Heal et al., 2009). We previously reported that intravenous d-amphetamine dose-dependently induces emergence from propofol and sevoflurane anesthesia in rats (Kenny et al., 2015). Consistent with a dopaminergic mechanism of action, we also found that administration of chloro-APB, a D_1_ dopamine receptor agonist, induces emergence from isoflurane anesthesia (Taylor et al., 2013). Because d-amphetamine promotes anesthetic emergence via dopaminergic stimulation, these studies suggest that d-amphetamine may be efficacious as a non-selective reversal agent for a variety of sedatives and anesthetics. However, propofol, isoflurane and sevoflurane act primarily by enhancing GABA_A_ receptor function (Brown et al., 2011), and it is unknown if d-amphetamine reverses the effects of other anesthetics that have non-GABAergic mechanisms of action.

Dexmedetomidine is an α_2_-adrenergic receptor agonist that inhibits the release of norepinephrine from the locus ceruleus, and causes excitation of neurons in the preoptic hypothalamus that induce NREM-like sleep and concomitant body cooling (Macdonald et al., 1991; Zhang et al., 2015; Hemmings et al., 2019; Ma et al., 2019). In humans, dexmedetomidine produces a sedated state resembling stage 3 NREM sleep (Akeju et al., 2018) and unconsciousness at high doses (Mantz et al., 2011; Akeju et al., 2016; Flukiger et al., 2018). Ketamine is an antagonist of the NMDA-type glutamate receptor, as well as other ion channels including the nicotinic acetylcholine receptor and hyperpolarization-activated cyclic nucleotide-gated channel 1 (HCN1) (Orser et al., 1997; Ho and Flood, 2004; Zhou et al., 2013). Although the α_2_-adrenergic receptor antagonist, atipamezole, is available for veterinary use to reverse the effects of dexmedetomidine (Pertovaara et al., 2005), at present there are no clinically available reversal agents for dexmedetomidine or ketamine in humans.

In this study we tested the hypothesis that d-amphetamine accelerates recovery of consciousness after dexmedetomidine or ketamine administration in adult male and female rats, using a blinded, randomized, crossover experimental design. Loss of righting (LOR) was used as a surrogate endpoint for loss of consciousness. In addition, rats with pre-implanted electrodes were used to record local field potentials (LFPs) from the prefrontal cortex (PFC) and electromyograms (EMGs) from the trapezius muscles, to assess changes in neurophysiology and muscle activity. Experiments were also conducted with the D_1_/D_5_ dopamine receptor antagonist, SCH-23390, to determine whether the arousal-promoting actions of d-amphetamine were mediated by D_1_/D_5_ receptors.

## Materials and Methods

### Animals

All studies were carried out in accordance with the recommendations in the Guide for the Care and Use of Laboratory Animals, National Institutes of Health, and were approved by the Massachusetts General Hospital Institutional Animal Care and Use Committee. All efforts were made to minimize animal suffering. Reporting of the animal research in this study complies with the ARRIVE guidelines (Kilkenny et al., 2010).

Adult Sprague-Dawley rats (Charles River Laboratories, Wilmington, MA, United States) were housed on a standard day-night cycle (lights on from 7 am to 7 pm) with ad libitum access to food and water. All experiments were conducted between 9 am and 5 pm after habituation to the housing facility. Male and female rats (*n* = 8 each) were used for behavioral experiments, and six additional male rats were implanted with skull screws and intracranial electrodes for neural recordings. Sample sizes were based on those we have used previously in similar rodent studies (Kenny et al., 2015; Moody et al., 2020). A minimum of 3 days of rest was provided between experiments.

### Drugs

Dexmedetomidine hydrochloride (Sun Pharma, India), ketamine hydrochloride (Henry Schein), dextroamphetamine sulfate (Sigma-Aldrich) and SCH-23390 (R(+)-7-Chloro-8-hydroxy-3-methyl-1-phenyl-2,3,4,5-tetrahydro-1H-3-benzazepine hydrochloride, Sigma-Aldrich) were used. All drugs were dissolved in sterile normal saline and administered with a syringe pump via an intravenous catheter placed in the lateral tail vein under brief isoflurane anesthesia. After placement of the tail vein IV catheter, each rat regained righting and returned to a normal level of activity for at least 10 min before experimentation. A heating pad was set to 37°C and placed under the animals to maintain normothermia.

### Experimental Design

Loss and recovery of righting (LOR and ROR) were used to define loss and recovery of consciousness, respectively. LOR was defined as not returning all four paws to the ground following placement in the lateral decubitus position. Time to emergence was defined as the time from onset of saline/d-amphetamine to the time when all four paws touched the ground.

The doses of dexmedetomidine (50 μg/kg IV over 10 min) and ketamine (50 mg/kg IV over 10 min) were selected because they reliably induced LOR in all animals without significant respiratory depression or risk of fatal overdose. The d-amphetamine doses (1 or 3 mg/kg IV over 2 min) were selected based on our previous study measuring dose-dependent reversal of sevoflurane anesthesia (Kenny et al., 2015). The dose of SCH-23390 (0.2 mg/kg IV) was selected because it provided D_1_/D_5_ dopaminergic receptor blockade without profound sedation (Taylor et al., 2013).

A randomized, blinded, crossover design was employed for all behavioral experiments (Supplementary Figure S1). In the first experiment, dexmedetomidine was administered, and the animal was placed in the lateral decubitus position on a heating pad. d-amphetamine or saline was then administered 5 min after the dexmedetomidine infusion. Eight rats (four males and four females) chosen at random were given d-amphetamine first, and the other eight rats were given saline first. In the second experiment, dexmedetomidine was administered, immediately followed by SCH-23390 or saline, and then d-amphetamine (1 mg/kg over 2 min). Eight rats (four males and four females) chosen at random were given SCH-23390 first, and the other eight rats were given saline first. After these experiments, the same protocol was repeated with ketamine in place of dexmedetomidine. Animals were euthanized by deep isoflurane anesthesia after all experiments were completed.

### Surgical Placement of Intracranial Electrodes

Adult male rats (425 ± 39 g) underwent electrode implant surgery under isoflurane anesthesia (1.5–2.5%). Animals were placed in a stereotaxic frame (Model 962, David Kopf Instruments) atop an electric heating pad. The core temperature was monitored using a rectal probe and continuously maintained within 37.0 ± 0.5°C. Ophthalmic ointment was applied to the eyes every 1–2 h. Craniotomies were made for extradural EEG electrodes over the PFC [3.0 mm anterior-posterior (AP), −2.5 mm medial-lateral (ML)], parietal cortex (PC; −6.0 mm AP, −4.5 mm ML), and cerebellum (CB; −10.5 mm AP, 0.0 mm ML). Ground screws (−10.5 mm AP, 2.4 mm ML) and five to six anchor screws were also placed. Additional craniotomies were made for LFP recordings from bilateral PFC [3.5 mm AP, ± 0.8 mm ML, -3.8 mm dorsal-ventral (DV)] and white matter (0.0 mm AP, 1.6 mm ML, −3.5 mm DV) as ground. Tetrodes made from insulated tungsten wires were dipped in an organic dye, 1,1′-Dioctadecyl-3,3,3′,3′-Tetramethylindocarbocyanine Perchlorate (DiIC18(3); Vybrant DiI Cell-Labeling Solution, Thermo Fisher Scientific) before insertion in the PFC bilaterally. DiI was used to highlight the tract for later histological analysis. For EEG and EMG electrodes, the coating at the tip was removed from pieces of insulated stainless steel and 7-strand stainless steel wires, respectively. EEG wires were hooked in each hole, and EMG wires were inserted in both sides of the trapezius muscles. All electrodes were fastened to a 16-channel electronic interface board (EIB) using small gold pins (EIB Small Pins, Neuralynx), and the board was fixed with dental acrylic. The skin was closed with absorbable sutures. Following surgery, animals received daily ketoprofen (4 mg/kg SC, Zoetis) for 2 days to provide analgesia, and at least 7 days were provided for recovery from surgery before conducting experiments.

### Local Field Potential Recordings and Analysis

Recordings began 15 min prior to the start of the dexmedetomidine or ketamine infusion and ended 15 min after ROR. Signals were continuously recorded with an Omniplex D Neural Data Acquisition System. Analog signals were amplified with a 1X gain 16-channel headstage (HST/16o25-GEN2-18P-2GP-G1, Plexon) and a Plexon MiniDigiAmp gain of 1,000. Signals were digitized with a sampling rate of 40 kHz with a Plexon MiniDigiAmp, digitally filtered (Bessel, four poles, 200 Hz cutoff), and downsampled to 1 kHz in OmniPlex Server.

Data analysis was performed in MATLAB R2018b (MathWorks). Spectral analysis was implemented with functions from the Chronux toolbox (Mitra and Bokil, 2008). Spectrograms of frontal EEG and LFPs were computed using the multitaper method. LFP data was included in group-level analyses only after confirmation of the correct probe location. The Chronux function “mtspecgramc” was used with a 30-s sliding window shifted every 15 s, time-bandwidth product TW = 3, and five tapers. Power values were converted to decibels.

One-minute periods were used to compute group-level power spectral densities (PSDs) from all animals during the awake baseline and specific times of interest. For the awake condition, a recording without spindle or slow-delta oscillations was selected based on visual inspection and used to compute the PSD. For the anesthetized condition, “post-LOR” and “post-drug” recordings were analyzed 3 min after the DEX/ketamine infusion and 10 min after the saline/d-amphetamine infusion, respectively. The Chronux function “mtspectrumc” was used with a half-bandwidth of 1 Hz and five tapers. For each animal, awake baseline periods and periods of interest were divided into non-overlapping 3-s trials, and the power values for each trial were calculated and converted to decibels. Power spectral densities were then pooled across all rats and resampled by a bootstrap procedure to determine the 95% confidence intervals (CI) of the median power. This process was repeated 1,000 times, and the 2.5th and 97.5th percentiles of the resulting distribution were identified.

### Histological Analysis

Electrode locations were confirmed via postmortem histological analysis. Animals were deeply anesthetized with 5% isoflurane until cessation of breathing occurred, then perfused with phosphate-buffered saline (1X) followed by 4% formalin (VWR International). Brains were stored in formalin for at least 24 h, after which 60 μm coronal sections were cut using a vibratome (VT 1000S, Leica Microsystems). Sections were mounted on slides with VECTASHIELD Mounting Medium with DAPI (4′, 6-diamidino-2-phenylindole) (H-1200, Vector Laboratories) and imaged with an Axio Imager M2 fluorescence microscope (Zeiss). The tracks created by the tetrodes were identified by DiI staining.

### Statistical Analysis

Based on the D’Agostino and Pearson normality test, dexmedetomidine behavioral data violated parametric assumptions of normality, thus the data were transformed using aligned rank transformation and analyzed by two-way mixed factor ANOVA. Descriptive statistics are reported as medians and interquartile ranges (IQR). Bonferroni adjusted pairwise post-hoc contrasts were conducted on the aligned rank transformed data in a full factorial design. Percentile bootstrapped confidence intervals around the mean differences are reported. Nonparametric analyses were conducted in R 3.6.1 and R-studio using the ARTool package to estimate the mixed model.

Ketamine behavioral data adhered to parametric assumptions of normality and were analyzed using parametric methods. Bonferroni adjusted pairwise post-hoc contrasts were conducted on the least square means in a full factorial design, and difference scores are presented using 95% CIs. Sensitivity models were conducted that examined main effects and interactions related to sex differences (e.g., sex x condition). The analyses were conducted in R 3.4 and R-Studio using the lme4 package to estimate the mixed models. Where appropriate, all inferences are two-tailed with *p* < 0.05 interpreted for statistical significance.

## Results

### D-Amphetamine Promotes Rapid Recovery from Dexmedetomidine-Induced Unconsciousness that is Inhibited by the D_1_/D_5_ Receptor Antagonist SCH-23390

**FIGURE 1:**
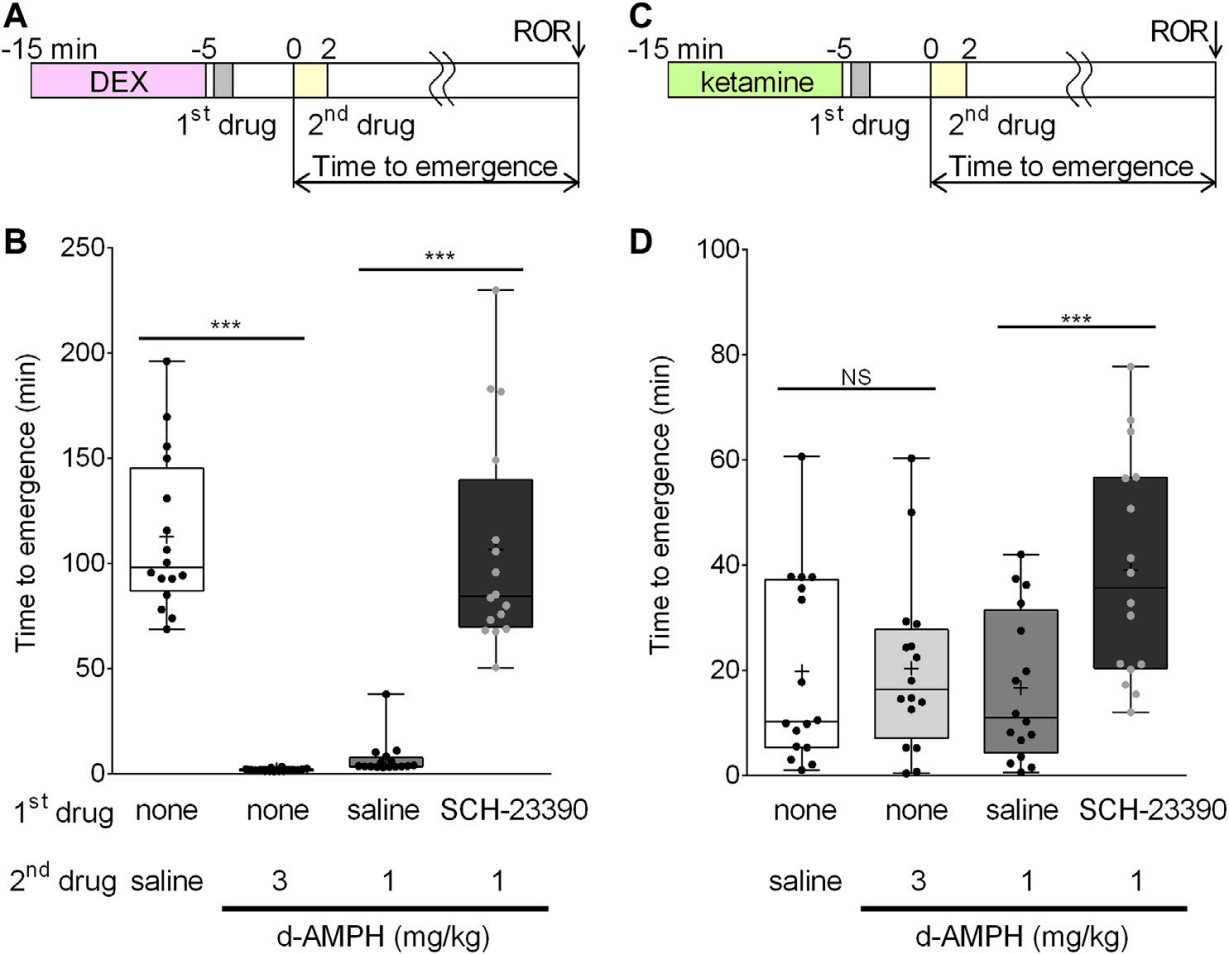
D-amphetamine accelerates recovery from unconsciousness induced by dexmedetomidine, but not ketamine. **(A)** Time course of the behavioral experiments with dexmedetomidine. The onset of the second drug infusion is set as time = 0. **(B)** Box-whisker plot of the time to emergence in all 16 rats after dexmedetomidine (50 μg/kg), followed by the listed drugs (plus sign = mean time to emergence, filled circle = individual data point). **(C)** Time course of the behavioral experiments with ketamine. The onset of the second drug infusion is set as time = 0. **(D)** Box-whisker plot of the time to emergence in all 16 rats after ketamine (50 mg/kg), followed by the listed drugs (***p < 0.001; Bonferroni post-hoc comparison; N.S. = not significantly different).

To assess the arousal effects of d-amphetamine and the D_1_/D_5_ dopamine receptor antagonist, SCH-23390, on dexmedetomidine recovery, four drug conditions were tested in a randomized, repeated measures design. Because the data failed tests of normality, nonparametric tests were used. The time course of this experiment is shown in Figure 1A. Dexmedetomidine (50 μg/kg over 10 min) induced LOR in all rats; however, median time to emergence from dexmedetomidine significantly differed based on drug condition (F (3.42) = 78.9, *p* < 0.0001) (Figure 1B). Bonferroni post-hoc comparisons revealed that d-amphetamine (3 mg/kg over 2 min, median emergence time = 1.8, IQR [1.3, 2.2]) reduced mean time to emergence by 111.0 min (95% CI [93.7, 130.6], *p* < 0.0001) compared to saline (median emergence time = 98.0, IQR [87.0, 145.1]). As 11/16 animals had ROR before the d-amphetamine infusion was complete, a lower dose of d-amphetamine (1 mg/kg) was used for subsequent experiments with dexmedetomidine. Pre-treatment with saline followed by 1 mg/kg d-amphetamine resulted in a median emergence time of 3.7 (IQR [3.4 7.7]), which was not significantly different from the 3 mg/kg d-amphetamine condition. The arousal effect of d-amphetamine (1 mg/kg) was blocked by pretreatment with 0.2 mg/kg SCH-23390 (median emergence time = 84.4, IQR [69.8, 139.5]), increasing the mean time to emergence by 99.7 min (95% CI [76.9, 126.9], *p* < 0.0001) (Figure 1B).

### Recovery From Ketamine-Induced Unconsciousness is Unaffected by D-Amphetamine and is Prolonged by D_1_/D_5_ Receptor Antagonism

Similar experiments were conducted with ketamine to test the hypothesis that d-amphetamine (3 mg/kg over 10 min) accelerates recovery from ketamine-induced unconsciousness. The time course of this experiment is shown in Figure 1C. Ketamine (50 mg/kg over 10 min) induced LOR in all rats. There was a significant main effect of treatment condition on mean time to emergence (F (4,59) = 17.7, *p* < 0.0001) (Figure 1D). The mean time to emergence was 19.7 ± 18.0 min after saline and 20.3 ± 16.5 min after d-amphetamine. Post-hoc comparisons revealed that this difference was not significant (95% CI [−9.7, 10.8], *p* = 1.000, Figure 1D).

To test the effect of administering a D_1_/D_5_ receptor antagonist prior to d-amphetamine, ketamine (50 mg/kg) was first administered, followed by SCH-23390 (0.2 mg/kg) or saline (vehicle), and then d-amphetamine (1 mg/kg). SCH-23390 followed by d-amphetamine (1 mg/kg) increased time to emergence by 22.4 min (95% CI [12.2, 32.6], *p* < 0.0001) compared to saline and d-amphetamine (1 mg/kg).

### Female Rats are More Sensitive to Dexmedetomidine and Ketamine-Induced Unconsciousness

**FIGURE 2:**
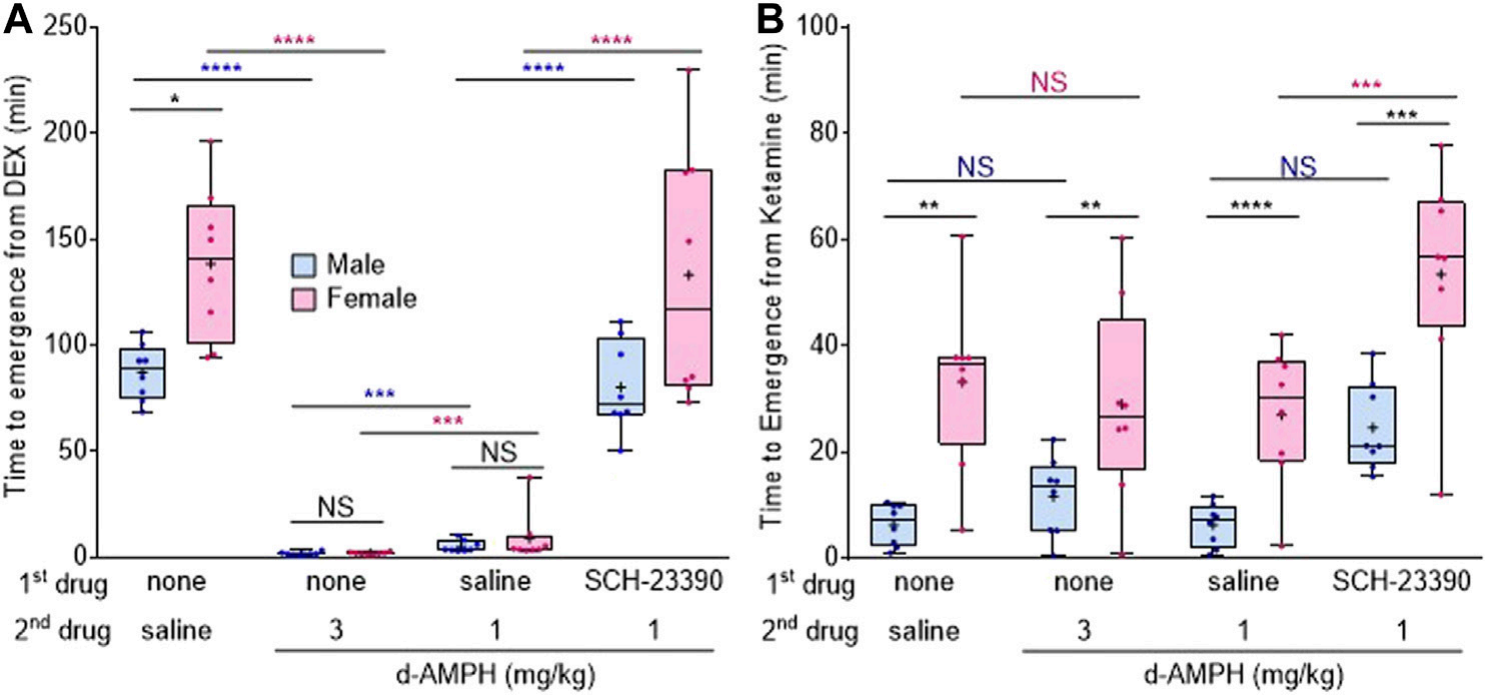
Female rats are more sensitive to dexmedetomidine- and ketamine-induced unconsciousness than males. **(A)** Box-whisker plot of the time to emergence in male (blue) and female (magenta) rats after dexmedetomidine (50 μg/kg), followed by the listed drugs (plus signs = mean time to emergence, filled circle = individual data point). **(B)** Box-whisker plot of the time to emergence after ketamine (50 mg/kg), followed by the listed drugs (*p < 0.05, **p < 0.01, ***p < 0.001, ****p < 0.0001, Bonferroni post-hoc comparison; N.S. = not significant; blue asterisk = comparison between males, magenta asterisk = comparison between females, black asterisk = between male and female comparison).

The rapid reversal of dexmedetomidine-induced unconsciousness by d-amphetamine was observed in both sexes. However, analysis revealed a significant interaction between sex and drug treatment on dexmedetomidine recovery (F_Condition x sex_ (3,42) = 5.7, *p* = 0.0024). After saline, female rats took 51.2 min longer (95% CI [26.1, 75.5], *p* = 0.0345) to emerge from dexmedetomidine than male rats (Figure 2A); however, these sex differences were lost when rats were treated with either 3 mg/kg d-amphetamine (mean difference 0.2 min, 95% CI [−0.7, 0.4], *p* = 0.9984) or 1 mg/kg d-amphetamine (mean difference 3.9 min, 95% CI [−2.0, 12.4], *p* = 0.9955).

Sex differences were also assessed under ketamine. Analysis revealed a main effect of sex on time to emergence (F_sex_ (1.16) = 75.8, *p* < 0.0001), with females overall taking longer to emerge than males, regardless of treatment condition. Bonferroni post-hoc comparisons revealed that male time to emergence from ketamine was not significantly affected by any treatment condition (Figure 2B). In contrast, SCH-23390 treatment with d-amphetamine (1 mg/kg) significantly increased female time to emergence compared to saline and 1 mg/kg d-amphetamine (95% CI [3.8, 49.2], *p* = 0.0076, Figure 2B).

### D-Amphetamine Induces Awake-Like Neurophysiology After Dexmedetomidine

**FIGURE 3:**
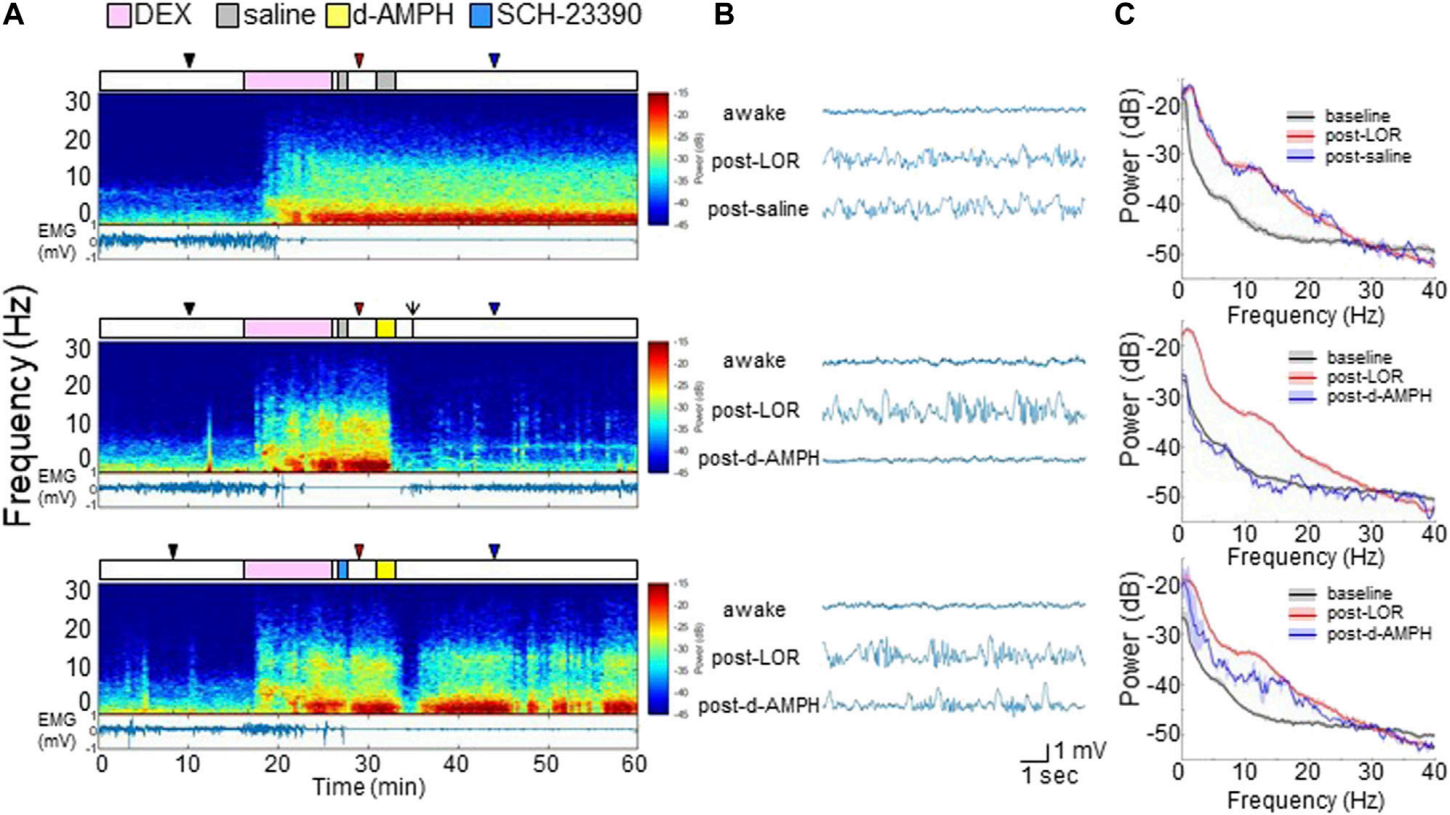
D-amphetamine restores awake-like neurophysiology in the PFC after dexmedetomidine. **(A)** Experimental time courses, spectrograms, and EMG recordings from a representative rat. The arrow indicates ROR. **(B)** Raw LFP waveforms from each time period. **(C)** The median and 95% CI of power spectral density during awake (black), post-LOC (red), and 10 min after saline or d-amphetamine (blue).

In six additional male rats, electrodes were implanted in the PFC to record LFPs (Supplementary Figure S2). Figure 3A shows representative spectrograms computed from PFC LFP recordings, and the EMG, from a representative rat that received different drug combinations (top: dexmedetomidine followed by two saline infusions; middle: dexmedetomidine followed by saline and then d-amphetamine; bottom: dexmedetomidine followed by SCH-23390 and then d-amphetamine). The experimental time courses are presented over the spectrograms. Dexmedetomidine broadly increased LFP spectral power at frequencies <25 Hz, decreased EMG activity, and induced LOR that was sustained for >30 min when saline was administered. When d-amphetamine was administered after dexmedetomidine, there was a broad and rapid decrease in LFP spectral power which coincided with return of EMG activity and ROR. After dexmedetomidine and SCH-23390, d-amphetamine only caused transient decreases in LFP spectral power that coincided with minimal EMG activity and no ROR.

Representative PFC LFP waveforms are shown in Figure 3B. Data are sampled from each time point indicated by the triangles (black: awake, red: post-LOR, blue: post-drug). Dexmedetomidine induced high-amplitude slow-delta (<4 Hz) oscillations after LOR, which remained after saline administration (top). D-amphetamine restored an awake-like LFP pattern with low amplitude and higher frequency (middle), but pretreatment with SCH-23390 largely inhibited this change (bottom).

Figure 3C shows power spectral density computed across all six rats during each period with median and 95% CI. As shown in the top panel, compared to the awake state (black), dexmedetomidine broadly increased power at frequencies <25 Hz (red) and the power spectrum did not change appreciably after saline (blue). However, as shown in the middle panel, d-amphetamine (blue) restored a power spectrum similar to the awake baseline (black). After SCH-23390 (bottom) d-amphetamine (blue) attenuated power at most frequencies <25 Hz, but did not fully restore the power spectrum back to the awake state (black).

### D-Amphetamine Does Not Induce Significant Changes in Neurophysiology After Ketamine

**FIGURE 4:**
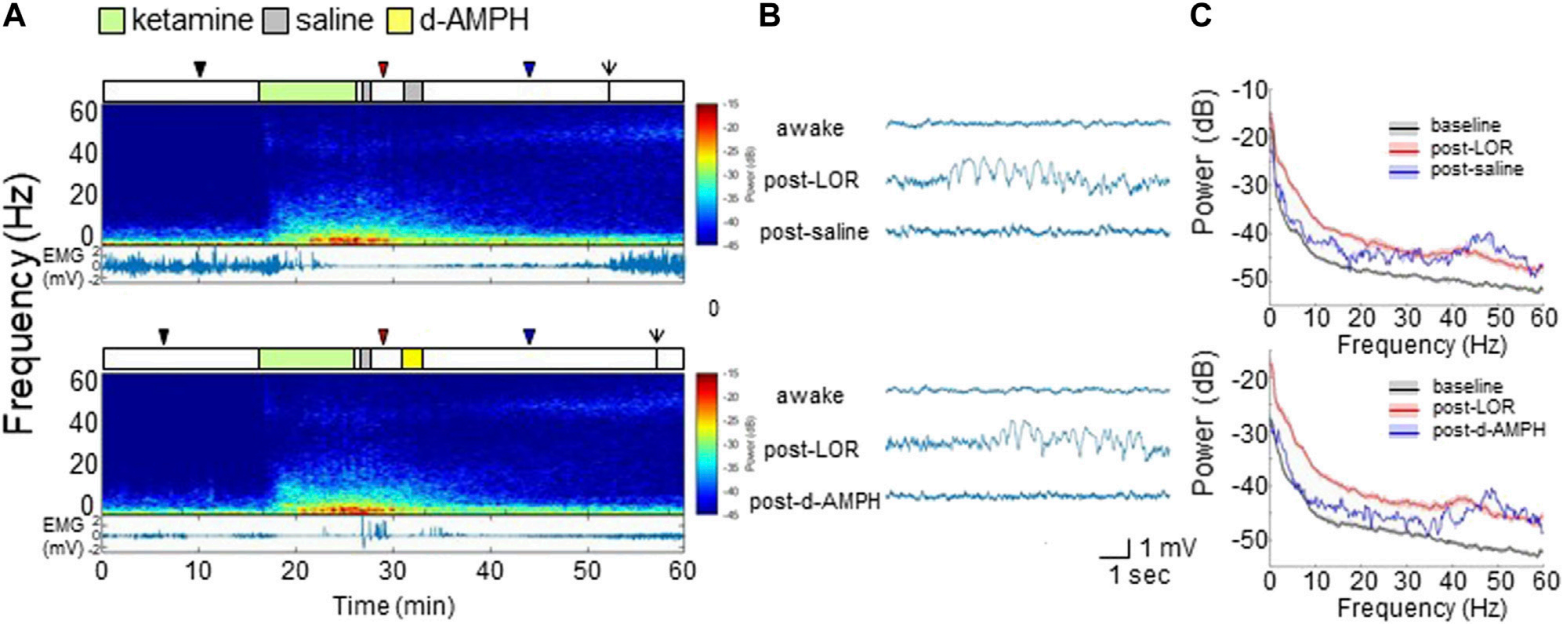
D**-**amphetamine does not appreciably alter the spectral content of PFC LFP recordings after ketamine. **(A)** Experimental time courses, spectrograms, and EMG recordings from a representative rat. The arrows indicate ROR. **(B)** Raw LFP waveforms from each time period. **(C)** The median and 95% CI of spectral density during awake (black), post-LOR (red), and 10 min after saline or d-amphetamine (blue).

Figure 4A shows representative spectrograms and EMG from a representative rat with different drug combinations (top: ketamine followed by two saline infusions; bottom: ketamine followed by saline and then d-amphetamine). Ketamine broadly increased LFP spectral power, decreased EMG activity, and induced LOR. Ketamine also increased γ (>40 Hz) power that remained elevated even after ROR. Compared to saline, d-amphetamine did not alter the spectrogram appreciably.

Representative PFC LFP waveforms are shown in Figure 4B. Compared to the awake state, ketamine induced high-amplitude slow-delta oscillations after LOR that gradually returned to a low-amplitude rhythm after saline (top). The waveform after d-amphetamine was qualitatively similar (bottom).

Figure 4C shows power spectral density computed across all rats during each period with median and 95% CI. As shown in the top panel of Figure 4C, compared to the awake state (black), ketamine broadly increased power up to 60 Hz (red). After saline (blue), there was decreased power <30 Hz, and increased power >45 Hz. As shown in the bottom panel of Figure 4C, d-amphetamine produced changes that were essentially indistinguishable from saline.

## Discussion

We found that d-amphetamine rapidly reduces time to emergence after dexmedetomidine. PFC LFP recordings revealed that dexmedetomidine broadly increases power at frequencies <25 Hz, and d-amphetamine promptly restores awake-like neurophysiology that coincides with increased EMG activity and ROR. These effects are inhibited by pretreatment with SCH-23390, demonstrating that they are mediated by D_1_ and/or D_5_ receptors. However, d-amphetamine did not accelerate recovery from ketamine, and did not affect ketamine-induced neurophysiology.

We previously reported that d-amphetamine induces emergence from propofol and sevoflurane anesthesia (Kenny et al., 2015), and that activation of D_1_ receptors induces emergence from isoflurane (Taylor et al., 2013). Additional studies revealed that electrical and optogenetic stimulation of dopaminergic neurons in the ventral tegmental area (VTA) induces emergence from isoflurane anesthesia (Solt et al., 2014; Taylor et al., 2016). Because d-amphetamine promotes dopamine release (Heal et al., 2009), these studies suggest that VTA dopaminergic neurons are chiefly responsible for anesthetic reversal by d-amphetamine. Inhibition of this effect by a D_1_/D_5_ antagonist provides further support for this hypothesis.

Recent studies have revealed that arousal-promoting neural circuits and neurotransmitters can accelerate recovery from anesthetic-induced unconsciousness (Kelz et al., 2019). In rats, administration of a cholinergic agonist in the PFC induces emergence from sevoflurane anesthesia (Pal et al., 2018). In humans, the cholinesterase inhibitor physostigmine restores consciousness from propofol anesthesia (Meuret et al., 2000). Caffeine, an adenosine receptor antagonist that increases cAMP and inhibits phosphodiesterase, accelerates emergence from isoflurane anesthesia in rodents and humans (Wang et al., 2014; Fong et al., 2018).

Orexin is an arousal-promoting neuropeptide that plays an important role in anesthetic emergence (Kelz et al., 2008; Zhang et al., 2012). In rodents, selective activation of orexin neurons via designer receptors shortens time to emergence from isoflurane anesthesia (Zhou et al., 2018). More recently, it was reported that optogenetic activation of orexinergic neurons projecting to the VTA promotes emergence from isoflurane anesthesia (Li et al., 2019), suggesting that part of the orexin-mediated arousal effect is mediated by VTA dopaminergic neurons.

Dexmedetomidine elicits sedative, analgesic, anxiolytic, and neuroprotective effects via the α_2_ adrenergic receptor (Bol et al., 1997; Hunter et al., 1997; Lakhlani et al., 1997). α_2_ antagonists such as atipamezole and yohimbine inhibit dexmedetomidine-induced sedation (Scheinin et al., 1998; Nelson et al., 2003), but these drugs are not available for human use. However, d-amphetamine is approved for human use and the present findings suggest that an intravenous formulation may be efficacious as a reversal agent for dexmedetomidine. This approach to dexmedetomidine reversal may find utility in both the perioperative and critical care settings. Furthermore, the availability of a reversal agent may allow for higher doses of dexmedetomidine to be used.

In male rats, the elimination half-lives of amphetamine and dexmedetomidine are ∼5–9 h (Kuhn and Schanberg, 1978) and ∼1 h (Bol et al., 1997), respectively. The duration of LOR that we observed in male rats was consistent with the findings of Bol et al. In humans, the elimination half-lives of d-amphetamine and dexmedetomidine are ∼10 and ∼2 h, respectively (accessdata.fda.gov). Therefore, in both rodents and humans the arousal effects of d-amphetamine would be expected to outlast the sedating effects of dexmedetomidine.

In addition to reversal of propofol and sevoflurane anesthesia (Kenny et al., 2015), we recently reported that d-amphetamine accelerates recovery from respiratory depression and unconsciousness induced by fentanyl (Moody et al., 2020). These findings suggest that d-amphetamine may be clinically useful to reverse the effects of multiple anesthetics and sedatives that have distinct mechanisms of action. However, the cardiovascular side effects of d-amphetamine (e.g., hypertension and tachycardia) may limit its utility in the clinical setting.

In contrast, d-amphetamine did not accelerate recovery from ketamine anesthesia. Depletion of arousal-promoting noradrenergic neurons in the locus coeruleus shortens the duration of ketamine anesthesia (Kushikata et al., 2011), indicating that modulation of arousal-promoting neurons can have paradoxical effects with ketamine. Furthermore, ketamine increases dopamine in the PFC via AMPA/kinate receptors (Moghaddam et al., 1997; Lorrain et al., 2003), and increase norepinephrine in the medial PFC (Kubota et al., 1999). These studies and our present findings suggest that enhancing noradrenergic and dopaminergic neurotransmission is ineffective to accelerate recovery from ketamine anesthesia.

We recorded from the PFC because this brain region is homologous to the human medial PFC (Hoover and Vertes, 2007). In humans, dexmedetomidine increases EEG δ (1–4 Hz), θ (4–8 Hz), and α (8–12 Hz) power in the frontal regions of the brain, while β (12–30 Hz) power decreases (Akeju et al., 2014; Akeju et al., 2016). In this rodent study, dexmedetomidine broadly increased PFC LFP power at all frequencies <25 Hz. While the neurophysiological effects of dexmedetomidine are qualitatively similar in humans and rodents, the differences observed in β power may be due to species differences and/or differences in relative dosing.

Ketamine also induced broad power increases in the PFC LFP, although the magnitude was smaller than dexmedetomidine. In addition, γ power increased gradually after the ketamine infusion and remained elevated after ROR. MK-801, a selective NMDA antagonist, induces γ burst oscillations in the rat frontal cortex like ketamine (Hiyoshi et al., 2014), and ketamine also paradoxically decreases the time to emergence from isoflurane anesthesia, coinciding with high γ power (Hambrecht-Wiedbusch et al., 2017) as well as increased coherence and normalized symbolic transfer entropy between frontal and parietal regions (Li et al., 2017). In the present study, a significant difference was not observed in γ power between the two groups that received saline or d-amphetamine, and there were no statistically significant differences in mean times to ROR after saline and d-amphetamine administration under ketamine anesthesia.

Pharmacokinetics and pharmacodynamics can differ between sexes due to physiological differences such as total body fat, enzyme expression, and hormonal fluctuations (Buchanan et al., 2009; Soldin and Mattison, 2009). Interestingly, several human studies indicate that women recover from general anesthesia faster than men. In 274 adult patients under general anesthesia with a combination of propofol, alfentanil and nitrous oxide, women emerged faster than men despite comparable anesthetic dosing (Gan et al., 1999). In 463 adults undergoing elective inpatient surgery, women emerged significantly faster than men (Myles et al., 2001). In 1,079 patients undergoing general anesthesia with neuromuscular blockade, women were reported to have faster times to eye opening and meeting PACU discharge criteria (Buchanan et al., 2006). The mechanisms underlying these sex differences have not been established.

In the present study, time to emergence after dexmedetomidine and ketamine was longer in females and varied widely compared to males. It has been reported after low-dose ketamine that female rats exhibit a slower clearance rate and longer half-life compared to males (Saland and Kabbaj, 2018) which may explain, at least in part, the prolonged duration of ketamine hypnosis that we observed in females. Similar pharmacokinetic data is not available for dexmedetomidine, but it is possible that such differences also account for the prolonged duration of dexmedetomidine hypnosis observed in female rats. The slowest emerging females differed among testing days, making it unlikely that body fat and other stable physiological characteristics are responsible for this variability. Alternatively, the hormonal changes that occur during the estrus cycle may influence anesthetic sensitivity and time to emergence.

Despite the sex differences in recovery times, d-amphetamine profoundly accelerated emergence times for both sexes after dexmedetomidine, whereas it had no effect on either sex after ketamine. Estrogen has long been known to affect the dopaminergic system, including dopamine release (Becker, 1990), turnover rates (Di Paolo et al., 1985), receptor sensitivity (Di Paolo et al., 1988), and reuptake (Morissette and Di Paolo, 1993). Whether endogenous hormone fluctuations in female rats influences sensitivity to anesthetic-induced unconsciousness warrants further exploration.

One limitation of this study is the small sample size. While experiments were originally powered to detect the robust d-amphetamine effects we have previously reported (Kenny et al., 2015), the unexpected presence of an interaction between sex and drug conditions prompted deeper analysis of the data. Knowledge of these sex differences will better inform future experimental design, allowing more subtle differences to be investigated.

In conclusion, d-amphetamine may be clinically useful to rapidly reverse dexmedetomidine-induced unconsciousness by promoting dopamine release and stimulating D_1_ and/or D_5_ receptors in the brain. Because d-amphetamine also accelerates recovery of consciousness after propofol, sevoflurane and fentanyl, an intravenous formulation may be an efficacious reversal agent for a variety of sedatives and anesthetics, with the notable exception of ketamine.

## Data Availability

The original contributions presented in the study are included in the article/Supplementary Material, further inquiries can be directed to the corresponding author.

## Abbreviations

LOR, loss of righting reflex; ROR, recovery of righting reflex; PFC, prefrontal cortex; EEG, electroencephalogram; EMG, electromyogram; LFP, local field potential.
