# Supplementary material for: D-Amphetamine Rapidly Reverses Dexmedetomidine-Induced Unconsciousness in Rats
Source: Front Pharmacol. 2021 May 18;12:668285. doi: 10.3389/fphar.2021.668285 (PMC8167047; doi:10.3389/fphar.2021.668285)
Supplement: Supplementary file 1 [file Image2.pdf]

Supplemental Figure 2

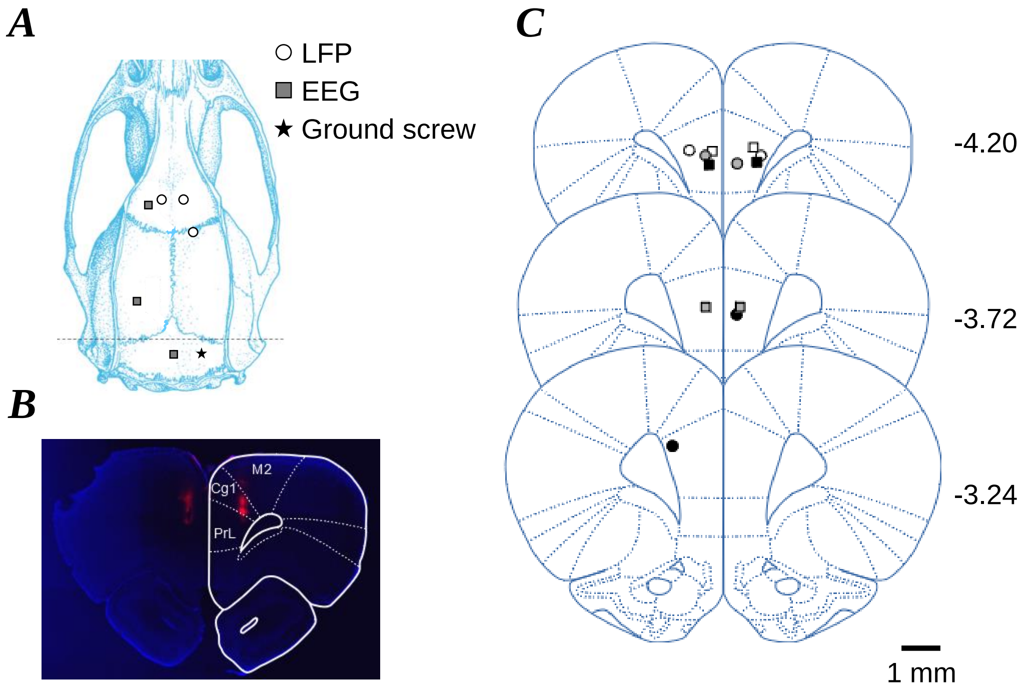

Histological analysis of LFP electrode locations.
A. Schematic of targeted sites over a rat skull. Symbols represent locations where LFP (white circles) and EEG electrodes (gray squares, prefrontal cortex, parietal cortex, and cerebellum), and ground screws (black star) were placed. ***B.*** A representative coronal section mounted with DAPI. Probe locations are confirmed by DiI. ***C.*** Locations of the LFP electrodes in all 6 rats. LFP electrodes were inserted in bilaterally in the PFC, and each symbol indicates the probe location from each rat. Numbers indicate anterior-posterior position relative to bregma. Tetrodes in the right PFC spanned 4.2–3.7mm AP, 0.4–1.0mm ML, and 3.4–3.9mm DV, and in the left PFC spanned 4.2–3.2mm AP, -0.2 to -1.3 mm ML, and 3.1–3.8mm DV. PrL, prelimbic cortex; Cg1, cingulate cortex area 1; M2, secondary motor cortex.
