# Supplementary figures and images for: D-Amphetamine Rapidly Reverses Dexmedetomidine-Induced Unconsciousness in Rats
Source: Front Pharmacol. 2021 May 18;12:668285. doi: 10.3389/fphar.2021.668285 (PMC8167047; doi:10.3389/fphar.2021.668285)

Supplemental Figure 1

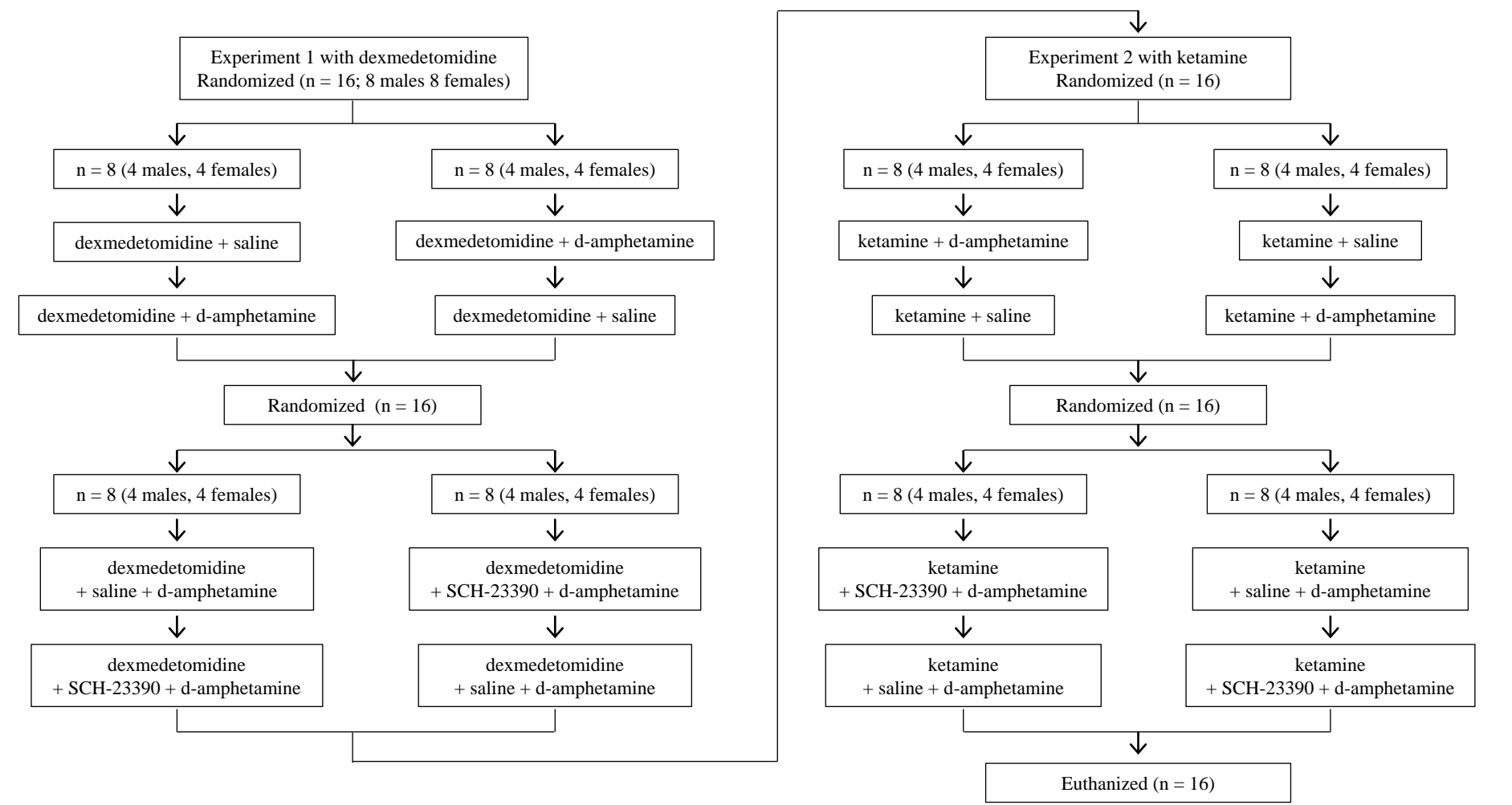

Flow-chart of behavioral experiments

Supplement: Supplementary file 2 [file Image1.pdf]
